# The complete mitochondrial genome of *Sarcophaga tsinanensis* (Diptera: Sarcophagidae)

**DOI:** 10.1080/23802359.2021.1923424

**Published:** 2021-09-06

**Authors:** Xiangrong Li, Tingjun Chen, Xiangyan Zhang, Li Yang, Xiaoling Pan, Yong Wang

**Affiliations:** a Department of Forensic Science, School of Basic Medical Sciences, Central South University, Changsha, Hunan, China; b Research Center for Special Medicine, Central South University, Changsha, Hunan, China; c The Key Laboratory of Model Animals and Stem Cell Biology in Hunan Province (2019TP1035), School of Medicine, Hunan Normal University, Changsha, Hunan, China

**Keywords:** Mitochondrial genome, *Sarcophaga tsinanensis*, phylogenetic analysis

## Abstract

*Sarcophaga tsinanensis* (Fan, 1964) (Diptera: Sarcophagidae), a species from subgenus *Heteronychia*. In this study, we report the complete mitochondrial genome (mitogenome) of *S. tsinanensis*. The length of this mitogenome was 14,972 bp in total, containing 13 protein-coding genes, 2 ribosomal RNAs, 22 transfer RNAs, and a non-coding control region. The arrangement of genes was the same as that of ancestral metazoan. *Sarcophaga tsinanensis* has a high nucleotide bias in A/T accounting for 76.80% of total nucleotides (A 39.9%, G 9.2%, C 13.9%, and T 36.9%). The result of phylogenetic analysis revealed that *S. tsinanensis* cluster together with species from the same subgenus *Heteronychia*, showing a clear monophyletic relationship. *Sarcophaga tsinanensis* is closely related to its sister species *Sarcophaga depressifrons*, *Sarcophaga plotnikovi*, and *Sarcophaga shnitnikovi*. This study provides the mitochondrial data of *S. tsinanensis* for further research on evolutionary relationships and species identification of flesh flies.

Sarcophagidae is a family of flies with forensic significance, which can provide valuable data for the estimation of minimum postmortem interval (PMImin) and other crime scene related information (Ren et al. 2018). Subgenus *Heteronychia* was the largest lineage of *Sarcophaga* (Diptera: Sarcophagidae) in species number (Daniel et al. 2013). At present, only a few species from subgenus *Heteronychia* has been discovered and studied (Krčmar et al. 2019; Ren et al. 2020). In this study, we presented the complete mitochondrial genome (mitogenome) of *Sarcophaga tsinanensis* (Fan 1964), which could further enrich our understanding of the phylogenetic relationship and phylogeography of subgenus *Heteronychia*.

In our study, adult specimens of *S. tsinanensis* were first trapped by decomposing pig livers in July 2020 in Beijing city (40°22′N, 116°23′E), China. All specimens were processed by freezing, and then identified based on traditional morphological features (Xu and Zhao 1996). All specimens were stored at −80 °C in Dr. Wang’s Lab (Changsha, Hunan, China) with a unique code (CSU20200713). Total DNA was extracted from thoracic muscle tissues using TIANamp Micro DNA Kit (Tiangen Biotech Co., Ltd, Beijing, China) according to the manufacture’s instruction. The sequencing of *S. tsinanensis* mitogenome was carried out with an Illumina HiSeq 2500 Platform (paired-end 150 bp) at OE Biotech. Co., Ltd. (Shanghai, China). *de novo* assembly was performed using MITObim v1.9 and SOAPdenovo v2.04 (https://github.com/chrishah/MITObim and http://soap.genomics.org.cn/soapdenovo.html) (Hahn et al. 2013). Finally, the preliminary annotation of all genes was determined by MITOS2 Web Server (http://mitos2.bioinf.uni-leipzig.de/index.py) (Bernt et al. 2013). To ensure the accuracy of the annotation, 13 protein coding genes (PCGs) were compared with published Sarcophagidae mitoticgenomes using MEGA X (Kumar et al. 2018), The transfer RNAs (tRNAs) were predicted by tRNAscan-SE Search Server v1.21 (Lowe and Chan 2016) and verified by comparison with those from other dipteran insects.

The length of *S. tsinanensis* mitogenome was 14,972 bp in total (GenBank no. MW423621), containing 13 PCGs, 2 ribosomal RNAs (rRNAs), 22 tRNAs, and a non-coding control region. The arrangement of genes was the same as those of ancestral metazoan (Cameron 2014). Among them, 9 PCGs and 14 tRNAs were encoded by H-strand, and the remaining 4 PCGs, 8 tRNAs and 2 rRNAs were encoded by L-strand. There was highly A/T bias in nucleotide composition, accounting for 76.80% of total nucleotides (A 39.9%, G 9.2%, C 13.9%, and T 36.9%). Maximum-likelihood (ML) analysis was performed in IQ-TREE v.1.6.8 with ultrafast likelihood bootstrap set to 1000 replicates (Nguyen et al. 2015). Phylogenetic analysis of *S. tsinanensis* and other 20 flesh flies were conducted using the ML method based on 13 PCGs of mitogenome, with *Chrysomya pinguis* (Diptera: Calliphoridae) as an outgroup (Figure 1). These 20 flesh fly species come from three genera. Two species belong to *Oxysarcodexia* genera, two species belong to *Boettcheria* genera, and all other species come from the same subgenus where *S. tsinanensis* belongs. All these sequences were aligned using MAFFT version 7 software (Katoh and Standley 2013).

**Figure 1.**
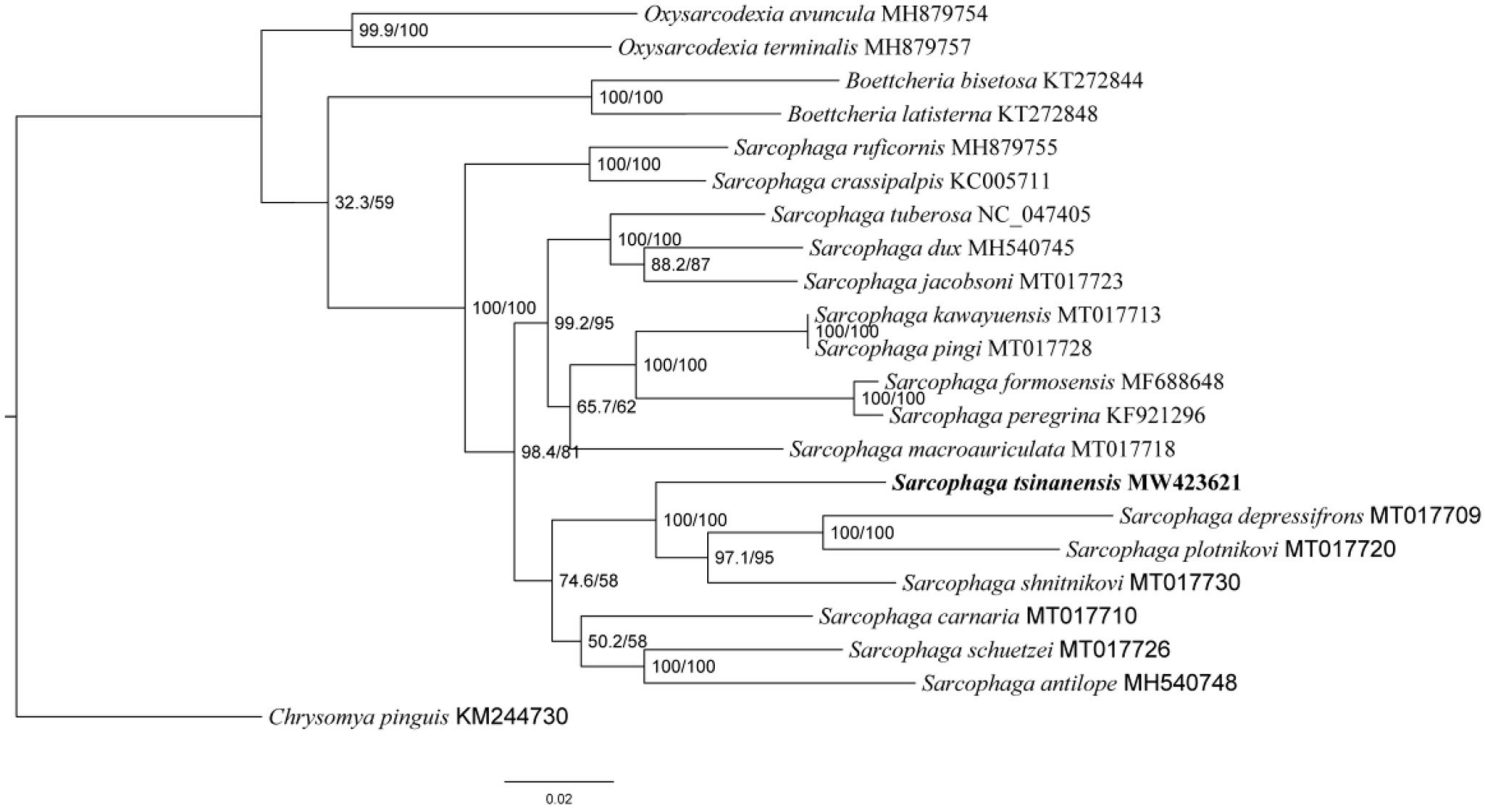
Phylogenetic trees of *S. tsinanensis* with other 20 flesh flies based on 13 protein-coding genes using the maximum-likelihood method. *Chrysomya pinguis* was selected as an outgroup.

The result of phylogenetic analysis revealed that *S. tsinanensis* clustered together with species from the same subgenus *Heteronychia*, showing a clear monophyletic relationship, and *S. tsinanensis* is closely related to its sister species *Sarcophaga depressifrons*, *Sarcophaga plotnikovi*, and *Sarcophaga shnitnikovi*. Since there are only limited mitogenomes of *Heteronychia* subgenus currently available, this study provides mitochondrial data of *S. tsinanensis* for further research on evolutionary relationships and species identification of flesh flies.

## Data Availability

The assembled mitochondrial genome is available on NCBI at https://www.ncbi.nlm.nih.gov/nuccore/MW423621 (Accession no. MW423621). Associated BioProject, SRA, and BioSample accession numbers are https://www.ncbi.nlm.nih.gov/bioproject/PRJNA690669/, https://www.ncbi.nlm.nih.gov/sra/SRR13441657, and https://www.ncbi.nlm.nih.gov/biosample/SAMN17319966/, respectively.
